# Activins as Dual Specificity TGF-β Family Molecules: SMAD-Activation via Activin- and BMP-Type 1 Receptors

**DOI:** 10.3390/biom10040519

**Published:** 2020-03-29

**Authors:** Oddrun Elise Olsen, Hanne Hella, Samah Elsaadi, Carsten Jacobi, Erik Martinez-Hackert, Toril Holien

**Affiliations:** 1 Department of Clinical and Molecular Medicine, NTNU – Norwegian University of Science and Technology, 7491 Trondheim, Norway; 2 Department of Hematology, St. Olav’s University Hospital, 7030 Trondheim, Norway; 3 Novartis Institutes for BioMedical Research Basel, Musculoskeletal Disease Area, Novartis Pharma AG, CH-4056 Basel, Switzerland; 4 Department of Biochemistry and Molecular Biology, Michigan State University, East Lansing, MI 48824, USA

**Keywords:** activin, SMAD, BMP, ALK2, ACVR1, signal transduction

## Abstract

Activins belong to the transforming growth factor (TGF)-β family of multifunctional cytokines and signal via the activin receptors ALK4 or ALK7 to activate the SMAD2/3 pathway. In some cases, activins also signal via the bone morphogenetic protein (BMP) receptor ALK2, causing activation of the SMAD1/5/8 pathway. In this study, we aimed to dissect how activin A and activin B homodimers, and activin AB and AC heterodimers activate the two main SMAD branches. We compared the activin-induced signaling dynamics of ALK4/7-SMAD2/3 and ALK2-SMAD1/5 in a multiple myeloma cell line. Signaling via the ALK2-SMAD1/5 pathway exhibited greater differences between ligands than signaling via ALK4/ALK7-SMAD2/3. Interestingly, activin B and activin AB very potently activated SMAD1/5, resembling the activation commonly seen with BMPs. As SMAD1/5 was also activated by activins in other cell types, we propose that dual specificity is a general mechanism for activin ligands. In addition, we found that the antagonist follistatin inhibited signaling by all the tested activins, whereas the antagonist cerberus specifically inhibited activin B. Taken together, we propose that activins may be considered dual specificity TGF-β family members, critically affecting how activins may be considered and targeted clinically.

## 1. Introduction

The transforming growth factor (TGF)-β family consists of more than 30 members in humans, including TGF-βs, bone morphogenetic proteins (BMPs), activins and growth differentiation factors (GDFs) [1]. The ligands mainly signal through two canonical pathways of SMAD transcription factors, but there is also compelling evidence that non-canonical activation can occur when a ligand–receptor signaling complex is formed [2]. The determining factor for which specific SMAD pathway will relay the signal is which type 1 receptor is activated in the signaling complex [3]. The SMAD2/3 pathway is activated by the type 1 receptors ALK4, ALK5 and ALK7, whereas the SMAD1/5/8 pathway is activated by the type 1 receptors ALK1, ALK2, ALK3 and ALK6 [3]. The approved HUGO (Human Genome Organisation) gene names for TGF-β family receptors are provided in Supplementary Table S1 for reference. The TGF-βs signal through ALK5 and activate the SMAD2/3 pathway but have also been shown to form complexes with ALK1 and/or ALK2 and activate the SMAD1/5/8 pathway. BMPs mostly form complexes with ALK1, ALK2, ALK3 or ALK6 and activate the SMAD1/5/8 pathway. Activins activate the SMAD2/3 pathway through the type 1 activin receptors ALK4 and ALK7 [4,5]. Activin A was originally found to interact with ALK2, although no activation of the SMAD1/5/8 pathway was detected in these studies [4,6,7]. It was also reported that activin A could act as an antagonist of BMP signaling by competing for receptor binding [8,9,10,11]. Recently, it has become clear that activin A and activin B also activate SMAD1/5 through mutated and wild type ALK2 [11,12,13,14,15]. Knockdown of BMPR2 caused potentiation of activin-induced SMAD1/5, but not SMAD2/3, activity in multiple myeloma and hepatocytic carcinoma cells [14,16]. We have shown that BMPs induce growth arrest and/or apoptosis in myeloma cells via activation of the SMAD1/5 pathway which in turn leads to c-MYC downregulation [17,18]. Maintaining c-MYC levels is important for the survival of myeloma cells [19]. We also reported that some myeloma cells are sensitive to activin A- and activin B-induced apoptosis via ALK2-SMAD1/5 [16]. Together, these studies suggest that activins are dual specificity TGF-β family ligands that can activate both SMAD branches, depending on the context.

Here, we aimed to characterize and compare activation of both SMAD branches by activins. Using an activin-responsive myeloma cell line, IH-1, we compared the effects of activin A, B, and C homo- and heterodimers on signaling dynamics and amplitude. We then used small molecule inhibitors with different specificities to further establish the involvement of different receptors in both IH-1 myeloma cells and HepG2 liver carcinoma cells. We further transiently knocked down type I receptors to establish receptor dependencies and compared the effects of the well-known activin antagonist follistatin and the less characterized activin B antagonist cerberus on downstream activation of SMAD2/3 and SMAD1/5 signaling. Although our results show that activins can act as BMP antagonists, we also find that in certain contexts activins may be considered dual specificity members of the TGF-β family. In these cases, ALK2 relays the signal from activins to SMAD1/5.

## 2. Materials and Methods 

### 2.1. Cells and Reagents

In this study, we used the human multiple myeloma cell lines IH-1 and INA-6. IH-1 was established in our laboratory [20]. INA-6 cells were a kind gift from Dr. M. Gramatzki (University of Erlangen-Nurnberg, Erlangen, Germany) [21]. IH-1 cells were cultured in 10% human serum (HS) (Department of Immunology and Transfusion Medicine, St. Olav’s University Hospital, Trondheim, Norway) in RPMI-1640 (Sigma-Aldrich Norway, Oslo, Norway) supplemented with 2 mM L-glutamine (Sigma-Aldrich), hereafter termed RPMI, whereas INA-6 cells were cultured in 10% fetal calf serum (FCS) in RPMI. For both myeloma cell lines, interleukin (IL)-6 (Gibco, Thermo Fisher Scientific, Waltham, MA, USA) (1 ng/mL) was added to the growth medium. The hepatocyte carcinoma cell line HepG2 was from European Collection of Authenticated Cell Cultures (ECACC; Salisbury, UK). HepG2 cells were grown in 10% FCS in Eagle’s minimum essential medium (EMEM) supplemented with 2 mM glutamine and non-essential amino acids (Sigma-Aldrich), hereafter termed EMEM. The cells were maintained in 37 °C in a humidified atmosphere containing 5% CO_2_ and tested regularly for mycoplasma. For experiments with myeloma cell lines, 2% HS in RPMI was used as the medium with IL6 (1 ng/mL) added for INA-6, and for HepG2, 0.1% bovine serum albumin (BSA) in EMEM was used. The recombinant human proteins activin B, activin C, and activin AC were from R&D Systems (Bio-Techne, Abingdon, UK), whereas *E. coli*-produced and refolded activin A (human) [22] and activin AB (human/Xenopus) were kindly provided by Marko Hyvönen’s group at the University of Cambridge, UK. Recombinant human cerberus was expressed and purified as previously described [23]. Recombinant human TGF-β1 and TGFβRII-Fc were from R&D Systems. The inhibitors K02288 and SB431542 were from Sigma-Aldrich, ML347, LDN-193189 and RepSox (Compound 19) were from Selleck Chemicals, Houston, TX, USA, and ZC-47-C95 (resynthesized compound 18a published by Jin et al., 2011 [24]) was from Novartis.

### 2.2. Western Blotting

Treated cells were pelleted, washed once in ice-cold phosphate-buffered saline (PBS), and lysed in a buffer consisting of 1% IGEPAL^®^ CA-630 (Sigma-Aldrich), 50 mM Tris–HCl (pH 7.5), 150 mM NaCl, 10% glycerol, 1 mM Na_3_VO_4_, 50 mM NaF and Complete Mini protease inhibitor cocktail (Roche, Basel, Switzerland). The samples were denatured and separated on NuPAGE™ Bis-Tris polyacrylamide gels (Invitrogen, Thermo Fisher Scientific). The gels were then blotted onto 0.45-μm nitrocellulose membranes (Bio-Rad, Hercules, CA). The membranes were blocked with 5% non-fat dried milk in Tris-buffered saline with 0.1% Tween 20 (TBS-T) and incubated with primary antibodies as indicated at 4 °C. Detection was performed with horseradish peroxidase-conjugated secondary antibodies (DAKO Cytomation, Copenhagen, Denmark) and SuperSignal West Femto Maximum Sensitivity Substrate (Thermo Fisher Scientific). Images were acquired and analyzed with Odyssey FC and Image Studio Software (LI-COR Biosciences, Lincoln, NE, USA). The primary antibodies that were used were: phospho-SMAD1/5 (RRID: AB_491015, Cat# 9516), phospho-SMAD2 (RRID: AB_490941, Cat# 3108), phospho-SMAD2/3 (RRID: AB_2631089, Cat# 8828), and cleaved caspase-3 (RRID: AB_2070042, Cat# 9664) (Cell Signaling Technology, BioNordika AS, Oslo, Norway), c-MYC (RRID: AB_2148606, Cat# 551102, BD Biosciences, Trondheim, Norway), and glyceraldehyde-3-phosphate dehydrogenase (GAPDH) (RRID: AB_2107448, Cat# Ab8245, Abcam, Cambridge, UK). Antibodies were diluted 1:1000, except for GAPDH which was diluted 1:30000.

### 2.3. Cell Viability

Relative viable cell numbers were determined by the CellTiter-Glo assay (Promega, Madison, WI, USA), which measures ATP levels, as described [25].

### 2.4. Transfection of INA-6 Cells

INA-6 cells were transfected using the Nucleofector device (Amaxa Biosystems, Cologne, Germany) and Amaxa Cell Line Nucleofector Kit R (Lonza, Basel, Switzerland), as previously described [26]. The siRNAs used were ON-TARGETplus SMARTpool *ACVR1/ALK2*, siGENOME SMARTpool *ACVR1B/ALK4*, *ACVR1C/ALK7* and *TGFBR1/ALK5*, and Non-Targeting Pool (Dharmacon, Thermo Fisher Scientific)

### 2.5. Transfection of HepG2 Cells

HepG2 cells were seeded in 24-well plates and left over night to adhere. Cells at a confluence of about 50% were transfected with siRNA using Lipofectamine RNAiMAX (Invitrogen) according to the protocol. The siRNAs used were ON-TARGETplus SMARTpool *ACVR1/ALK2* and Non-Targeting Pool (Dharmacon). The day after transfection, the cells were treated with the indicated ligands for 1 h and harvested for western blotting or PCR.

### 2.6. Comparative RT-PCR

RNA was isolated using the RNeasy Mini Kit (Qiagen, Crawley, UK) and complementary DNA was synthesized from total RNA using the High Capacity RNA-to-cDNA kit (Applied Biosystems, Thermo Fisher Scentific). PCR was performed using StepOne Real-Time PCR System and Taqman Gene Expression Assays (Applied Biosystems) as described previously [9]. The Taqman assays used were: *ACVR1* (Hs00153836_m1), *ACVR1B* (Hs00244715_m1), *ACVR1C* (Hs0000899854_m1), *TGFB1* (Hs00998133_m1), and *GAPDH* (Hs99999905_m1). The comparative Ct method was used to calculate relative changes in gene expression with *GAPDH* as the housekeeping gene.

### 2.7. Statistical Analysis

GraphPad Prism 8 (Graphpad Software, Inc., San Diego, LA) was used to analyze statistical significance. The tests used for each experiment are described in the figure legends.

## 3. Results

### 3.1. Activin Dimers Have Dose-Dependent Effects on IH-1 Cell Viability

**Figure 1:**
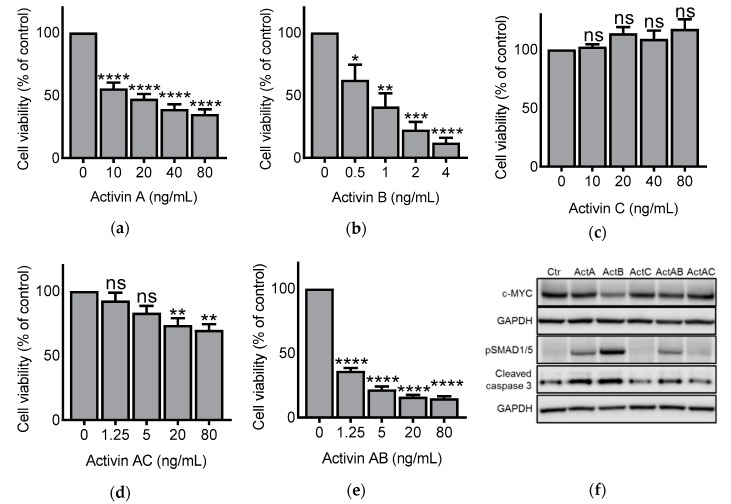
Impact of activin homo- and heterodimers on IH-1 cell viability. IH-1 myeloma cells were treated for three days with increasing doses of activins as indicated in the figure. Cell viability was measured using CellTiter Glo and the results are plotted relative to control (**a**–**e**). The graphs represent mean ± standard error of the mean (s.e.m.) of *n* = 3 independent experiments. One-way ANOVA and Dunnett’s multiple comparisons test were performed (* *p* ≤ 0.05, ** *p* ≤ 0.01, *** *p* ≤ 0.001, **** *p* ≤ 0.0001, ns (not significant) *p* > 0.05). (**f**) We also treated IH-1 cells for 24 h with activin A (20 ng/mL), activin B (4 ng/mL), activin C (20 ng/mL), activin AB (5 ng/mL) or activin AC (20 ng/mL), and did western blot to look at differences in expression of c-MYC, phospho-SMAD1/5 (pSMAD1/5) and cleaved caspase 3, with GAPDH as the loading control. The blots shown are representative of *n* = 3 independent experiments.

IH-1 myeloma cells were treated with activin hetero- and homodimers for three days before cell viability was determined by measuring ATP content in wells. As we have shown before, activin A and activin B dose-dependently reduced cell viability, with activin B being the most potent cell viability inhibitor (Figure 1a,b) [16]. As expected, no difference in cell viability was seen after treatment with activin C at the given doses (Figure 1c). Activin AB reduced cell viability to a similar extent as activin B (Figure 1d), whereas activin AC was less potent than the other activins (Figure 1e). We further confirmed that the effect of activins on cell number, at least partially, depended on apoptosis due to correlation with SMAD-induced c-MYC downregulation and caspase-3 cleavage (Figure 1f) [17]. 

### 3.2. Activins Activated SMAD1/5 and SMAD2 with Different Dynamics

**Figure 2:**
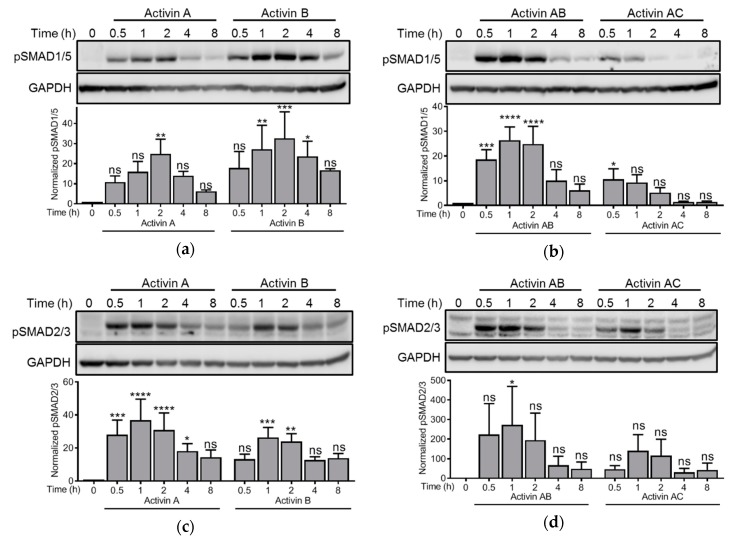
Time-series comparing activation of both SMAD branches by activins. IH-1 myeloma cells were treated with activin A (20 ng/mL), activin B (4 ng/mL), activin AB (5 ng/mL) or activin AC (20 ng/mL) for the indicated time-points. Then, the phosphorylation status of SMAD1/5 (**a**,**b**) or SMAD2/3 (**c**,**d**) was measured by western blot. Representative experiments are shown (upper panels) and relative activation was calculated based on signal intensities of the SMADs and GAPDH for normalization (lower panels). The graphs represent mean ± s.e.m. of *n* = 3 independent experiments. Two-way ANOVA and Bonferroni’s multiple comparisons test were performed (* *p* ≤ 0.05, ** *p* ≤ 0.01, *** *p* ≤ 0.001, **** *p* ≤ 0.0001, ns (not significant) *p* > 0.05).

We have previously shown that activin A and activin B activated SMAD1/5 via ALK2 and induced cell death in IH-1 and INA-6 myeloma cell lines [16]. Activation of the SMAD2/3 pathway did not lead to apoptosis in these cells, likely due to mechanisms that prevent translocation of activated SMAD2/3 to the nucleus in myeloma cells [9,16,27]. Extending on this finding, activation of SMAD1/5 by activin AB and activin AC also correlated with reduced cell viability (Figure 1c,d,f). Nevertheless, the activins activated both SMAD branches and we wanted to compare the signaling dynamics between these two. IH-1 cells were treated with activins and harvested for western blotting at different time points. Activin doses were chosen based on the viability assay and activin C was omitted in these experiments since we were not able to detect any activation of SMADs with this ligand (Figure 1c). Activation of the SMAD1/5 pathway peaked after 2 h for activin A and activin B, whereas it peaked after 1 h for activin AB, and as early as 0.5 h (or even earlier) for activin AC (Figure 2a,b). Activin AC was altogether not a strong inducer of SMAD1/5 activity and the activation was terminated quickly compared to the other activins. Interestingly, activation of SMAD2 was much more similar amongst the different activins and peaked at 1 h for all the tested ligands (Figure 2c,d).

### 3.3. Effect of Small Molecule Inhibitors on Activin-Induced SMAD Phosphorylation

**Figure 3:**
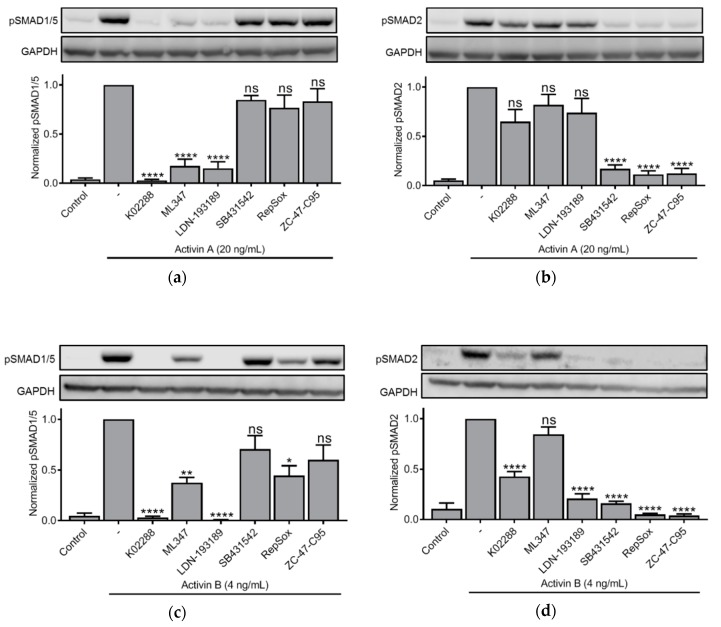
Effect of small molecule inhibitors on activin-induced SMAD phosphorylation. IH-1 myeloma cells were treated for one hour with activin A (20 ng/mL) (**a**,**b**) or activin B (4 ng/mL) (**c**,**d**) with or without different inhibitors. K02288, LDN-193189 and RepSox (100 nM), ML347 (200 nM), ZC-47-C95 (1 µM) and SB431542 (2 µM). Then, the phosphorylation status of SMAD1/5 and SMAD2 was measured by western blot. Representative experiments are shown (upper panels) and relative activation was calculated based on signal intensities of the SMADs and GAPDH for normalization (lower panels). The graphs represent mean ± s.e.m. of *n* = 3 independent experiments. One-way ANOVA and Dunnett’s multiple comparisons test were performed (* *p* ≤ 0.05, ** *p* ≤ 0.01, **** *p* ≤ 0.0001, ns (not significant) *p* > 0.05).

We next sought to characterize the type 1 receptor involved in activation of the SMAD-branches and used small molecule inhibitors. Such inhibitors are valuable tools, but their degree of specificity and sensitivity varies, so we chose to use six different inhibitors. The inhibitors K02288, ML347, and LDN-193189, are known inhibitors of the SMAD1/5/8-pathway via BMP type 1 receptors (e.g., ALK1, ALK2, ALK3 and ALK6) [28,29,30]. The inhibitors SB431542, RepSox, and ZC-47-C95 are described as inhibitors of the SMAD2/3 pathway via activin/TGF-β type 1 receptors (e.g., ALK4, ALK5 and ALK7) [24,31,32]. A table describing relative specificities of the inhibitors is shown (Supplementary Table S2). We treated IH-1 cells with activin A and combined activin A with the panel of inhibitors for comparison. As expected, activation of SMAD1/5 by activin A in these cells was dependent on a BMP type 1 receptor (Figure 3a). Activation of SMAD2 was, also as expected, dependent on an activin/TGF-β type 1 receptor (Figure 3b). For comparison we also tested activin B, and surprisingly, the panel of inhibitors showed less clear results (Figure 3c,d). For instance, ML347 was less efficient towards activin B-induced SMAD1/5 activation and RepSox showed some inhibiting activity (Figure 3c). Furthermore, in addition to the expected inhibition by activin/TGF-β type 1 receptor inhibitors towards activin B-induced SMAD2 activation, there was also significant inhibition by the BMP type 1 receptor inhibitors K02288 and LDN-193189. These results could indicate the dependency of a BMP type 1 receptor for activation of SMAD2 by activin B. Thus, based on this experiment, activation of SMAD1/5 by activin A seems to be entirely dependent on a BMP type 1 receptor in IH-1 cells, whereas there might be an interdependent relationship between the activation of the two SMAD-branches for activin B.

### 3.4. Effect of the BMP Type 1 Receptor Inhibitor, K02288, on Activin-Induced SMAD Activity in HepG2 Cells

**Figure 4:**
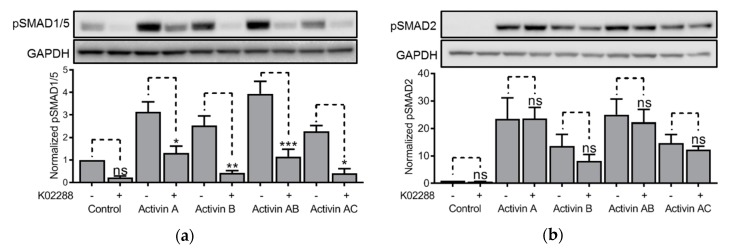
Effect of the bone morphogenic protein (BMP) type 1 receptor inhibitor, K02288, on activin-induced SMAD activity in HepG2 cells. HepG2 cells were treated for one hour with activin A, activin AB. Activin AC (20 ng/mL), or activin B (50 ng/mL) with or without K02288 (100 nM). Then, the phosphorylation status of SMAD1/5 (**a**) and SMAD2 (**b**) was measured by western blot. Representative experiments are shown (upper panels) and relative activation was calculated based on signal intensities of the SMADs and GAPDH for normalization (lower panels). The graphs represent mean ± s.e.m. of *n* = 3 independent experiments. Two-way ANOVA and Bonferroni’s multiple comparisons test were performed (* *p* ≤ 0.05, ** *p* ≤ 0.01, *** *p* ≤ 0.001, ns (not significant) *p* > 0.05).

We also detected activation of both SMAD-branches in activin-treated HepG2 liver carcinoma cells. In these cells, we applied the K02288 BMP type 1 receptor inhibitor and checked for effects of activin-induced SMAD1/5- and SMAD2-activation. In comparison to the IH-1 cells, the results were as expected, with inhibition of SMAD1/5, but not SMAD2 activation (Figure 4).

### 3.5. ALK2 Knockdown Blunted Activin-Induced SMAD1/5 Activity

**Figure 5:**
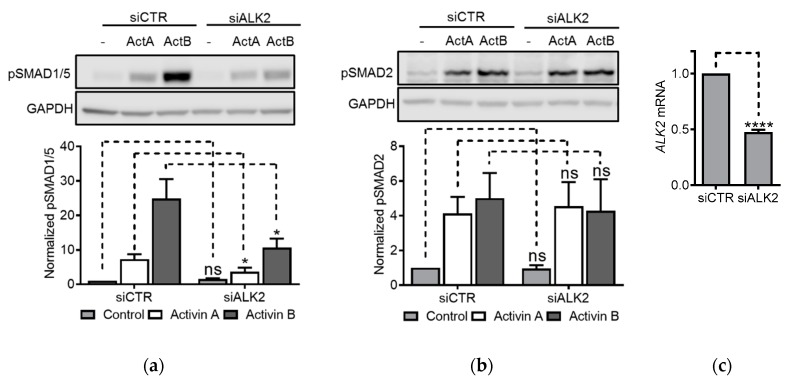
ALK2 knockdown blunted activin-induced SMAD1/5 activity. INA-6 myeloma cells were transfected with siRNA targeting ALK2 or Non-Targeting Pool as a control. The cells were treated 2 days post transfection with activin A (50 ng/mL) or activin B (10 ng/mL) for 2 h. Then, the phosphorylation status of SMAD1/5 (**a**) and SMAD2 (**b**) was measured by western blot. Representative experiments are shown (upper panels) and relative activation was calculated based on signal intensities of the SMADs and GAPDH for normalization (lower panels). The graphs represent mean ± s.e.m. of *n* = 3 independent experiments. Two-way ANOVA and Bonferroni’s multiple comparisons test were performed (* *p* ≤ 0.05, ns (not significant) *p* > 0.05). (**c**) *ALK2* mRNA levels were measured by PCR using the comparative Ct method and *GAPDH* as housekeeping gene. The graph represents mean ± s.e.m. of three independent experiments. A two-tailed, paired *t*-test was performed (**** *p* ≤ 0.0001).

Since the results using inhibitors only partially explained type 1 receptor usage, we chose a more specific approach to further clarify the mechanism of SMAD1/5 activation. We performed ALK2 knockdown experiments in another multiple myeloma cell line, INA-6, which expresses ALK2, but not ALK3 [16,25,33]. ALK2 siRNA or control siRNA were transfected into the cells. After two days, the cells were treated with activin A or activin B for 1 h and SMAD activity was measured by western blots. Knockdown of ALK2 significantly blunted both activin A- and activin B-induced SMAD1/5 phosphorylation but had no effect on SMAD2/3 phosphorylation (Figure 5a,b). The knockdown efficacy was just above 50%, as measured by PCR (Figure 5c). For comparison, we also transiently knocked down the expression of the other type 1 receptors ALK4, ALK7 and ALK5. As expected, none of these siRNAs affected activin A- and activin B-induced SMAD1/5 phosphorylation (Supplementary Figure S1). Moreover, in HepG2 cells, knockdown of ALK2 counteracted activation of SMAD1/5, but not SMAD2, after treatment with activin A or activin B (Supplementary Figure S2).

Multiple myeloma cells express high levels of TGF-β mRNA and protein [18]. To exclude the possibility that the SMAD activation following activin treatment was not caused by changes in autocrine TGF-β activity, we first measured *TGFB1* mRNA in IH-1 and INA-6 cells treated with activin A and activin B for 4 h (Supplementary Figure S3a,b). There were no significant changes in *TGFB1* expression levels. Then, we treated IH-1 and INA-6 cells with activin A, activin B or TGF-β, with or without soluble TGFβRII-Fc, an inhibitory TGF-β ligand trap and looked at activation of SMAD1/5 and SMAD2 (Supplementary Figure S3c–f). The soluble TGFβRII-Fc prevented TGF-β-induced SMAD activation but failed to inhibit activation of either of the two SMAD branches by activins. Similar results were seen with a neutralizing TGF-β antibody (data not shown).

### 3.6. Antagonism of Activins by Follistatin and Cerberus

**Figure 6:**
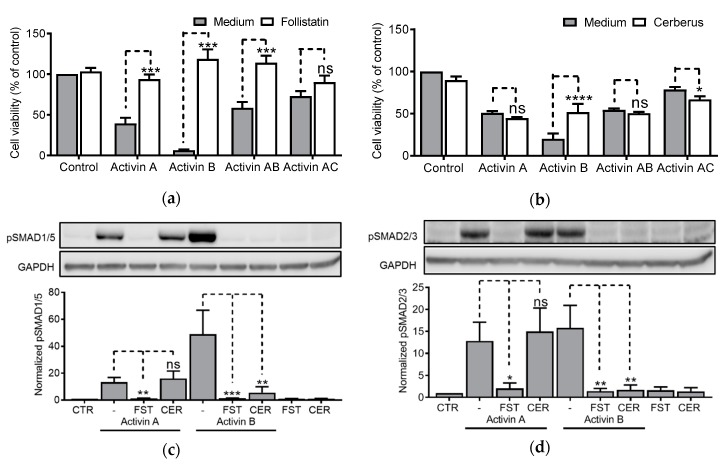
Antagonism of activins by follistatin and cerberus. IH-1 myeloma cells were treated for three days with activin A or activin AC (20 ng/mL), activin B (4 ng/mL) or activin AB (5 ng/mL) in combination with either (**a**) follistatin (1 µg/mL) or (**b**) cerberus (4 µg/mL) before cell viability was measured using CellTiter Glo. Results are plotted relative to the control. The phosphorylation status of SMAD1/5 (**c**) and SMAD2/3 (**d**) was measured by western blot. IH-1 myeloma cells were treated for one hour with activin A (20 ng/mL) or activin B (50 ng/mL) with or without follistatin (1 µg/mL) or cerberus (4 µg/mL). Representative experiments are shown (upper panels) and relative activation was calculated based on signal intensities of the SMADs and GAPDH for normalization (lower panels). The graphs (**a**–**c**) represent mean ± s.e.m. of *n* = 3 independent experiments. Two-way ANOVA and Bonferroni’s multiple comparisons test were performed (* *p* ≤ 0.05, ** *p* ≤ 0.01, *** *p* ≤ 0.001, **** *p* ≤ 0.0001, ns (not significant) *p* > 0.05).

Having shown that activins depend on ALK2 for activation of the SMAD1/5 pathway, we wanted to compare extracellular activin antagonists to see if activation of both SMAD branches was inhibited. Follistatin is an important activin antagonist, and the ratio of serum activin A/follistatin is commonly used as a measure of activin A bioavailability [34,35,36]. We treated IH-1 cells with activin dimers together with follistatin for three days before measuring relative cell viability. As expected, the growth inhibitory effect of all the tested activin dimers was blunted in the presence of follistatin, except for activin AC, where no significant effect was observed (Figure 6a). Cerberus is recognized as an antagonist of the TGF-β family ligand nodal [23,37]. Interestingly, human cerberus also binds and antagonizes activin B and, to a lesser extent, BMP6 and BMP7 [38]. As far as we know, activin AB and activin AC have never been tested for in vitro antagonism by human cerberus. We therefore treated IH-1 cells with activin dimers together with cerberus for three days before measuring relative cell viability. As expected, cerberus was found to specifically antagonize activin B, but did not antagonize activin A, activin AB or activin AC (Figure 6b). We confirmed using western blots that the effect on cell viability correlated with effect on SMAD activity (Figure 6c,d). Our results thus confirm and supplement previous studies showing that follistatin is a general activin antagonist, whereas cerberus can distinguish between activin B and the other activins.

## 4. Discussion

Activin A usually signals through ALK4, whereas activin B and activin AB bind and signal either through ALK4 or ALK7, resulting in activation of the SMAD2/3 pathway [5,39,40]. Activin C is thought to be non-signaling, and thereby inhibitory, by forming activin AC heterodimers at the expense of more potent activin A homodimers [41,42]. Here, we show that activins can activate signals via the two main branches of the SMAD signaling pathway, SMAD2/3 and SMAD1/5/8, by binding and activating different TGF-β family type I receptors. In addition, we directly compare the activity of four different activin homo- and heterodimers. We show that the signaling strength and dynamics induced by the different ligands vary. Importantly, this is to our knowledge the first time activin AB and activin AC have been shown to activate SMAD1/5 via the wildtype ALK2 receptor.

Interestingly, the activin AC heterodimer activates both SMAD branches, whereas activin C is inactive. Similarly, activin A is a weak activator of SMAD1/5, whereas the heterodimer activin AB is approximately as potent as activin B. Type I receptors have been shown to bind at the interface between the two protomers in the dimeric ligand [43,44]. Thus, our observations raise the possibility that only one of the ligand protomers is needed to activate ALK2 signaling.

Sequences both in the receptors and the ligand determine binding specificity and strength. Recent studies have suggested that the finger 2 tip loops in activin A and GDF11 are important for their type I receptor binding specificities [44,45]. Moreover, in some cell types, only mutated ALK2, such as the variant encoded by ACVR1 (R206H), has been shown to relay a signal to SMAD1/5 after activin binding [11,12]. Thus, there has been a debate about whether activins can activate SMAD1/5 via the wildtype ALK2 type 1 receptor. For instance, overexpressed wildtype ALK2 could relay activin A induced SMAD1/5 activity in immortalized mouse embryonic fibroblasts [15], but this was not seen in HEK cells [11]. However, in systems with overexpression of ALK2, the balance between type I receptors, type II receptors and co-factors may be altered in a way that precludes proper signaling. It was shown that activin B activated SMAD1/5 and thereby increased the levels of the SMAD-regulated target gene *HAMP* (encoding hepcidin) in hepatocytes [13]. By using siRNA, induction of *HAMP* by activin B was found to be dependent on ALK2, ACVR2A, ACVR2B, and to a lesser extent ALK3 [14]. We showed that activin A and activin B likely signal through endogenous wildtype ALK2 to activate SMAD1/5 in myeloma and hepatocellular cell lines and the present study further supports this finding [16]. We speculate that the activin-mediated activation of SMAD1/5 via wildtype ALK2 that occurs in some cell types depends on a co-factor. In hepatocytes, hemojuvelin, encoded by *HJV*, may be such a co-factor [14]. In myeloma cells, on the other hand, *HJV* is not expressed. Thus, involvement of another, unidentified co-factor is more likely and may warrant further investigation. 

Interestingly, in cases where activin-induced activation of the SMAD1/5 pathway is not seen, and also in cases where the activation of SMADs via ALK2 is weak, the activins may act as BMP antagonists rather than agonists [8,9,10,11]. For example, activin A binds with high affinity to the type II receptors ACVR2A and ACVR2B, and since these receptors are shared with BMPs, activin A can act as an antagonist of BMP9 [9,10]. Ligand competition as a way of regulating SMAD activity is an interesting concept that may greatly impact signaling outcomes in cells. 

TGF-β is another ligand which can activate both SMAD branches; however, the mechanism is likely different. TGF-β induces SMAD2/3 signaling via ALK5 in complex with the type II TGF-β receptor [46]. However, TGF-β also induces SMAD1/5 signaling in endothelial cells, likely via lateral activation of ALK1, which was found in the same heteromeric signaling complexes as ALK5 [47,48]. In epithelial cells, similar lateral signaling was shown for TGF-β dependent activation of SMAD1/5 via ALK2 and ALK3 [49,50]. In these cases, activation of the SMAD1/5 pathway was dependent on the kinase activity of ALK5. This is not the case for the activins, as we have shown before and confirm here [16]. Therefore, the concept of lateral TGF-β signaling is likely largely dependent on context. There is currently no evidence of lateral TGF-β induced activation of SMAD1/5 via ALK2 in myeloma cells. We showed here that TGF-β activated SMAD1/5 in INA-6, but not IH-1 cells. In contrast, we found that activin-induced SMAD1/5 phosphorylation in both cell lines was independent of TGF-β and ALK5. 

There were no huge differences in SMAD1/5 and SMAD2/3 signaling dynamics in the IH-1 cells. However, for activin A and activin B there was a delay (peak at 2 h versus 1 h) in SMAD1/5 activation compared to SMAD2/3 activation, suggesting a more indirect effect of these ligands on SMAD1/5 activation. However, the different signaling dynamics between these pathways in myeloma cells could also suggest that there are different receptor complexes that relay the signal to each of the SMAD branches, i.e., activins do not activate ALK2 and ALK4/ALK7 via a common heteromeric receptor complex. It is not known if mixed SMAD complexes, which include SMADs from both branches, are formed by activin treatment in these cells. Another explanation for the delay in SMAD1/5 activation compared with SMAD2/3 could be that signaling dynamics are affected by variations in the localization and internalization of the two pathways’ respective receptors, as was suggested for BMP4 and activin A [51]. In that study, the authors suggested that BMP4 treatment caused transient turnover of receptors, which resulted in oscillatory signaling dynamics. By contrast, activin A induced a depletion of receptors matched with receptor renewal, which resulted in a more stable signal. Differences between BMP4 and activin A signaling dynamics also contrasted with TGF-β, which caused a refractory state in cells upon acute stimulation [52]. The refractory state was likely caused by a rapid depletion of TGF-β receptors from the cell surface. Finally, there is also a possibility that the activated SMAD molecules may be terminated by dephosphorylation at different rates for each SMAD branch [53]. 

In this study, we used six different small molecule inhibitors of varying specificities. Results obtained with the inhibitors should be interpreted with caution, as there is both a degree of uncertainty with regards to the actual specificities of each inhibitor, and the promiscuity in the ligand–receptor system is not fully known. For inhibition of activin A, the results were clear and supported the conclusion that activin A induces SMAD1/5 signaling via wildtype ALK2. For activin B, however, the results were less clear. Firstly, ML347, the most specific ALK2 inhibitor, less potently inhibited activin B-induced SMAD1/5 activation than other inhibitors for this pathway (K02288 and LDN-193189). Notably, both K02288 and LDN-193189 also inhibit ALK3 (Supplementary Table S2). This result could possibly be a matter of dose and a more careful titration of each inhibitor could potentially give clearer results. Secondly, the SMAD1/5 activation inhibitors K02288 and LDN-193189, also inhibited SMAD2 phosphorylation in IH-1 myeloma cells treated with activin B. It was puzzling that this effect was observed for activin B, but not activin A. The results could indicate that activin B-induced SMAD2/3 activation benefits from the kinase activity of a type I BMP receptor, which in IH-1 cells could be either ALK2 or ALK3, or that these inhibitors also block the activin B dependent kinase ALK7 [16,33]. However, the same trend was not replicated in HepG2 cells when these cells were treated with K02288 or siRNA targeting ALK2. Furthermore, INA-6 cells only express ALK2, and not ALK3 or ALK6, and in these cells, knocking down ALK2 with siRNA did not counteract activin B induced SMAD2 activation. Thus, when considering all results, we propose that activin B induced SMAD2 activation does not require ALK2 kinase activity. However, we cannot rule out the possibility that the kinase activity of another type I BMP receptor, such as ALK3, positively affects SMAD2 activation by activin B. Indeed, it was shown that ALK3 and ALK2 can form BMP-dependent heterodimers that induce hepcidin expression in hepatocytes in vitro [54]. 

Follistatin (FST) is a well-known antagonist of activins and it was therefore not surprising to see that FST inhibited the activity of all the tested activins, both via the SMAD2/3 and the SMAD1/5 pathway. Cerberus’ ability to bind and antagonize activin B, but not activin A, has also been clearly described before [23,38]. Notably, although cerberus antagonized activin B induced signaling via both the SMAD2/3 pathway and the SMAD1/5 pathway, it did not antagonize activin AB heterodimers. Notably, although both follistatin and cerberus completely inhibited activin B induced SMAD activation, only follistatin fully rescued the effects of activin B on cell viability. The most important difference between the SMAD activation assay (1 h treatment) and the viability assay (three days of treatment) is time. In the duration of the viability assay, it is possible that cerberus fails to have a long-lasting antagonistic activity towards activin B, or cerberus may have an effect on other non-SMAD signaling pathways. This is a matter for future studies. 

## 5. Conclusions

**Figure 7:**
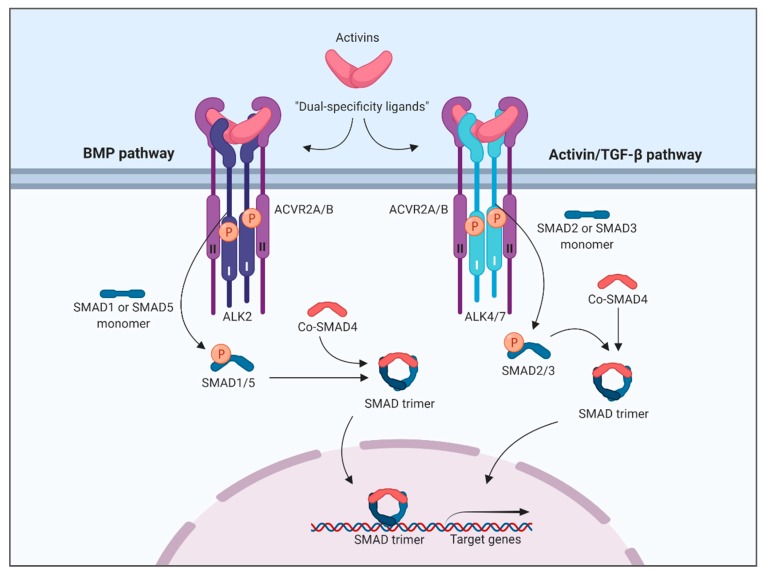
Proposed dual specificity of activins. Activins belong to the activin/TGF-β branch of the TGF-β family, but share the type II receptors ACVR2A and ACVR2B, and in some cases, BMPR2, with BMPs. The activins can therefore compete with BMPs for use of type II receptors. Usually, the type I receptor in the complex is either ALK4 or ALK7, and activation of these, lead to SMAD2/3 phosphorylation. Interestingly, activins may also form ligand–receptor complexes where the type I receptor is the BMP receptor ALK2. Activation of ALK2 leads to SMAD1/5 phosphorylation. The complex of activins, type II receptors and ALK2 can either be non-signaling (no SMAD1/5 phosphorylation), induce a weak activation of SMAD1/5, or potently activate SMAD1/5, depending on which activin is in the complex and the cellular context. This image was created with Biorender.com.

Activins act as dual-specificity TGF-β family molecules by engaging different type I receptors and activating both branches of the SMAD signaling pathway (Figure 7). Activation likely occurs in different ligand–receptor complexes, as we show that the signaling dynamics are different between the two SMAD branches. The dual specificity is dependent on context and we speculate that co-factors that are so far not known may be key to this context dependence. Importantly, in cases where activins are not able to activate the SMAD1/5 pathway, they may still occupy receptors that are shared with BMPs, thereby acting as antagonists of the BMP pathway. 

## Supplementary Materials

The following are available online at https://www.mdpi.com/2218-273X/10/4/519/s1, Figure S1: Effects of knockdown of activin/TGF-β type 1 receptors in INA-6 myeloma cells, Figure S2: Effects of ALK2 knockdown in HepG2 cells, Figure S3. Activin-induced SMAD activity was not caused by an autocrine TGF-β loop, Table S1: Approved HUGO gene names for TGF-β family receptors, Table S2. TGF-β/SMAD inhibitors; reported inhibitory potential.

## References

[1] Morikawa M., Derynck R., Miyazono K. (2016). TGF-beta and the TGF-beta Family: Context-Dependent Roles in Cell and Tissue Physiology. Cold Spring Harb. Perspect. Biol..

[2] Miyazono K., Kamiya Y., Morikawa M. (2010). Bone morphogenetic protein receptors and signal transduction. J. Biochem..

[3] Yadin D., Knaus P., Mueller T.D. (2016). Structural insights into BMP receptors: Specificity, activation and inhibition. Cytokine Growth Factor Rev..

[4] Ten Dijke P., Yamashita H., Ichijo H., Franzen P., Laiho M., Miyazono K., Heldin C.H. (1994). Characterization of type I receptors for transforming growth factor-beta and activin. Science.

[5] Tsuchida K., Nakatani M., Yamakawa N., Hashimoto O., Hasegawa Y., Sugino H. (2004). Activin isoforms signal through type I receptor serine/threonine kinase ALK7. Mol. Cell. Endocrinol..

[6] Attisano L., Carcamo J., Ventura F., Weis F.M., Massague J., Wrana J.L. (1993). Identification of human activin and TGF beta type I receptors that form heteromeric kinase complexes with type II receptors. Cell.

[7] Ebner R., Chen R.H., Lawler S., Zioncheck T., Derynck R. (1993). Determination of type I receptor specificity by the type II receptors for TGF-beta or activin. Science.

[8] Piek E., Afrakhte M., Sampath K., van Zoelen E.J., Heldin C.H., ten Dijke P. (1999). Functional antagonism between activin and osteogenic protein-1 in human embryonal carcinoma cells. J. Cell Physiol..

[9] Olsen O.E., Wader K.F., Hella H., Mylin A.K., Turesson I., Nesthus I., Waage A., Sundan A., Holien T. (2015). Activin A inhibits BMP-signaling by binding ACVR2A and ACVR2B. Cell Commun. Signal..

[10] Aykul S., Martinez-Hackert E. (2016). Transforming Growth Factor-beta Family Ligands Can Function as Antagonists by Competing for Type II Receptor Binding. J. Biol. Chem..

[11] Hatsell S.J., Idone V., Wolken D.M., Huang L., Kim H.J., Wang L., Wen X., Nannuru K.C., Jimenez J., Xie L. (2015). ACVR1R206H receptor mutation causes fibrodysplasia ossificans progressiva by imparting responsiveness to activin A. Sci. Transl. Med..

[12] Hino K., Ikeya M., Horigome K., Matsumoto Y., Ebise H., Nishio M., Sekiguchi K., Shibata M., Nagata S., Matsuda S. (2015). Neofunction of ACVR1 in fibrodysplasia ossificans progressiva. Proc. Natl. Acad. Sci. USA.

[13] Besson-Fournier C., Latour C., Kautz L., Bertrand J., Ganz T., Roth M.P., Coppin H. (2012). Induction of activin B by inflammatory stimuli up-regulates expression of the iron-regulatory peptide hepcidin through Smad1/5/8 signaling. Blood.

[14] Canali S., Core A.B., Zumbrennen-Bullough K.B., Merkulova M., Wang C.Y., Schneyer A.L., Pietrangelo A., Babitt J.L. (2016). Activin B Induces Noncanonical SMAD1/5/8 Signaling via BMP Type I Receptors in Hepatocytes: Evidence for a Role in Hepcidin Induction by Inflammation in Male Mice. Endocrinology.

[15] Haupt J., Xu M., Shore E.M. (2017). Variable signaling activity by FOP ACVR1 mutations. Bone.

[16] Olsen O.E., Sankar M., Elsaadi S., Hella H., Buene G., Darvekar S.R., Misund K., Katagiri T., Knaus P., Holien T. (2018). BMPR2 inhibits activin- and BMP-signaling via wild type ALK2. J. Cell Sci..

[17] Holien T., Vatsveen T.K., Hella H., Rampa C., Brede G., Groseth L.A., Rekvig M., Borset M., Standal T., Waage A. (2012). Bone morphogenetic proteins induce apoptosis in multiple myeloma cells by Smad-dependent repression of MYC. Leuk. Off. J. Leuk. Soc. Am. Leuk. Res. Fund UK.

[18] Holien T., Sundan A. (2014). The role of bone morphogenetic proteins in myeloma cell survival. Cytokine Growth Factor Rev..

[19] Holien T., Vatsveen T.K., Hella H., Waage A., Sundan A. (2012). Addiction to c-MYC in multiple myeloma. Blood.

[20] Hjertner O., Hjorth-Hansen H., Borset M., Seidel C., Waage A., Sundan A. (2001). Bone morphogenetic protein-4 inhibits proliferation and induces apoptosis of multiple myeloma cells. Blood.

[21] Burger R., Guenther A., Bakker F., Schmalzing M., Bernand S., Baum W., Duerr B., Hocke G.M., Steininger H., Gebhart E. (2001). Gp130 and ras mediated signaling in human plasma cell line INA-6: A cytokine-regulated tumor model for plasmacytoma. Hematol. J. Off. J. Eur. Haematol. Assoc..

[22] Harrington A.E., Morris-Triggs S.A., Ruotolo B.T., Robinson C.V., Ohnuma S., Hyvonen M. (2006). Structural basis for the inhibition of activin signalling by follistatin. Embo J..

[23] Aykul S., Ni W., Mutatu W., Martinez-Hackert E. (2015). Human Cerberus prevents nodal-receptor binding, inhibits nodal signaling, and suppresses nodal-mediated phenotypes. PLoS ONE.

[24] Jin C.H., Sreenu D., Krishnaiah M., Subrahmanyam V.B., Rao K.S., Nagendra Mohan A.V., Park C.Y., Son J.Y., Son D.H., Park H.J. (2011). Synthesis and biological evaluation of 1-substituted-3(5)-(6-methylpyridin-2-yl)-4-(quinoxalin-6-yl)pyrazoles as transforming growth factor-beta type 1 receptor kinase inhibitors. Eur. J. Med. Chem..

[25] Olsen O.E., Wader K.F., Misund K., Vatsveen T.K., Ro T.B., Mylin A.K., Turesson I., Stordal B.F., Moen S.H., Standal T. (2014). Bone morphogenetic protein-9 suppresses growth of myeloma cells by signaling through ALK2 but is inhibited by endoglin. Blood Cancer J..

[26] Fagerli U.M., Holt R.U., Holien T., Vaatsveen T.K., Zhan F., Egeberg K.W., Barlogie B., Waage A., Aarset H., Dai H.Y. (2008). Overexpression and involvement in migration by the metastasis-associated phosphatase PRL-3 in human myeloma cells. Blood.

[27] Baughn L.B., Di Liberto M., Niesvizky R., Cho H.J., Jayabalan D., Lane J., Liu F., Chen-Kiang S. (2009). CDK2 phosphorylation of Smad2 disrupts TGF-beta transcriptional regulation in resistant primary bone marrow myeloma cells. J. Immunol..

[28] Sanvitale C.E., Kerr G., Chaikuad A., Ramel M.C., Mohedas A.H., Reichert S., Wang Y., Triffitt J.T., Cuny G.D., Yu P.B. (2013). A new class of small molecule inhibitor of BMP signaling. PLoS ONE.

[29] Engers D.W., Frist A.Y., Lindsley C.W., Hong C.C., Hopkins C.R. (2013). Synthesis and structure-activity relationships of a novel and selective bone morphogenetic protein receptor (BMP) inhibitor derived from the pyrazolo[1.5-a]pyrimidine scaffold of dorsomorphin: The discovery of ML347 as an ALK2 versus ALK3 selective MLPCN probe. Bioorg. Med. Chem. Lett..

[30] Cuny G.D., Yu P.B., Laha J.K., Xing X., Liu J.F., Lai C.S., Deng D.Y., Sachidanandan C., Bloch K.D., Peterson R.T. (2008). Structure-activity relationship study of bone morphogenetic protein (BMP) signaling inhibitors. Bioorg. Med. Chem. Lett..

[31] Inman G.J., Nicolas F.J., Callahan J.F., Harling J.D., Gaster L.M., Reith A.D., Laping N.J., Hill C.S. (2002). SB-431542 is a potent and specific inhibitor of transforming growth factor-beta superfamily type I activin receptor-like kinase (ALK) receptors ALK4, ALK5, and ALK7. Mol. Pharmacol..

[32] Gellibert F., Woolven J., Fouchet M.H., Mathews N., Goodland H., Lovegrove V., Laroze A., Nguyen V.L., Sautet S., Wang R. (2004). Identification of 1,5-naphthyridine derivatives as a novel series of potent and selective TGF-beta type I receptor inhibitors. J. Med. Chem..

[33] Ro T.B., Holt R.U., Brenne A.T., Hjorth-Hansen H., Waage A., Hjertner O., Sundan A., Borset M. (2004). Bone morphogenetic protein-5, -6 and -7 inhibit growth and induce apoptosis in human myeloma cells. Oncogene.

[34] Nakamura T., Takio K., Eto Y., Shibai H., Titani K., Sugino H. (1990). Activin-binding protein from rat ovary is follistatin. Science.

[35] Schneyer A., Schoen A., Quigg A., Sidis Y. (2003). Differential binding and neutralization of activins A and B by follistatin and follistatin like-3 (FSTL-3/FSRP/FLRG). Endocrinology.

[36] Muenster U., Harrison C.A., Donaldson C., Vale W., Fischer W.H. (2005). An activin-A/C chimera exhibits activin and myostatin antagonistic properties. J. Biol. Chem..

[37] Piccolo S., Agius E., Leyns L., Bhattacharyya S., Grunz H., Bouwmeester T., De Robertis E.M. (1999). The head inducer Cerberus is a multifunctional antagonist of Nodal, BMP and Wnt signals. Nature.

[38] Aykul S., Martinez-Hackert E. (2016). New Ligand Binding Function of Human Cerberus and Role of Proteolytic Processing in Regulating Ligand-Receptor Interactions and Antagonist Activity. J. Mol. Biol..

[39] Wakefield L.M., Hill C.S. (2013). Beyond TGFbeta: Roles of other TGFbeta superfamily members in cancer. Nat. Rev. Cancer.

[40] Attisano L., Wrana J.L., Montalvo E., Massague J. (1996). Activation of signalling by the activin receptor complex. Mol. Cell. Biol..

[41] Mellor S.L., Cranfield M., Ries R., Pedersen J., Cancilla B., de Kretser D., Groome N.P., Mason A.J., Risbridger G.P. (2000). Localization of activin beta(A)-, beta(B)-, and beta(C)-subunits in humanprostate and evidence for formation of new activin heterodimers of beta(C)-subunit. J. Clin. Endocrinol. Metab..

[42] Mellor S.L., Ball E.M., O’Connor A.E., Ethier J.F., Cranfield M., Schmitt J.F., Phillips D.J., Groome N.P., Risbridger G.P. (2003). Activin betaC-subunit heterodimers provide a new mechanism of regulating activin levels in the prostate. Endocrinology.

[43] Kirsch T., Sebald W., Dreyer M.K. (2000). Crystal structure of the BMP-2-BRIA ectodomain complex. Nat. Struct. Biol..

[44] Goebel E.J., Corpina R.A., Hinck C.S., Czepnik M., Castonguay R., Grenha R., Boisvert A., Miklossy G., Fullerton P.T., Matzuk M.M. (2019). Structural characterization of an activin class ternary receptor complex reveals a third paradigm for receptor specificity. Proc. Natl. Acad. Sci. USA.

[45] Idone V., Corpina R., Goebel E., Cunanan C., Dimitriou A., Kim H., Zhang Q., Rafique A., Leidich R., Wang X. (2019). The finger 2 tip loop of Activin A is required for the formation of its non-signaling complex with ACVR1 and type II Bone Morphogenetic Protein receptors. bioRxiv.

[46] Heldin C.H., Moustakas A. (2016). Signaling Receptors for TGF-beta Family Members. Cold Spring Harb. Perspect. Biol..

[47] Goumans M.J., Valdimarsdottir G., Itoh S., Rosendahl A., Sideras P., ten Dijke P. (2002). Balancing the activation state of the endothelium via two distinct TGF-beta type I receptors. Embo J..

[48] Goumans M.J., Valdimarsdottir G., Itoh S., Lebrin F., Larsson J., Mummery C., Karlsson S., ten Dijke P. (2003). Activin receptor-like kinase (ALK)1 is an antagonistic mediator of lateral TGFbeta/ALK5 signaling. Mol. Cell.

[49] Daly A.C., Randall R.A., Hill C.S. (2008). Transforming growth factor beta-induced Smad1/5 phosphorylation in epithelial cells is mediated by novel receptor complexes and is essential for anchorage-independent growth. Mol. Cell. Biol..

[50] Ramachandran A., Vizan P., Das D., Chakravarty P., Vogt J., Rogers K.W., Muller P., Hinck A.P., Sapkota G.P., Hill C.S. (2018). TGF-beta uses a novel mode of receptor activation to phosphorylate SMAD1/5 and induce epithelial-to-mesenchymal transition. eLife.

[51] Miller D.S.J., Schmierer B., Hill C.S. (2019). TGF-beta family ligands exhibit distinct signalling dynamics that are driven by receptor localisation. J. Cell Sci..

[52] Vizan P., Miller D.S., Gori I., Das D., Schmierer B., Hill C.S. (2013). Controlling long-term signaling: Receptor dynamics determine attenuation and refractory behavior of the TGF-beta pathway. Sci. Signal..

[53] Bruce D.L., Sapkota G.P. (2012). Phosphatases in SMAD regulation. Febs Lett..

[54] Traeger L., Gallitz I., Sekhri R., Baumer N., Kuhlmann T., Kemming C., Holtkamp M., Muller J.C., Karst U., Canonne-Hergaux F. (2018). ALK3 undergoes ligand-independent homodimerization and BMP-induced heterodimerization with ALK2. Free Radic. Biol. Med..

