# Supplementary material for: Activins as Dual Specificity TGF-β Family Molecules: SMAD-Activation via Activin- and BMP-Type 1 Receptors
Source: Biomolecules. 2020 Mar 29;10(4):519. doi: 10.3390/biom10040519 (PMC7225989; doi:10.3390/biom10040519)
Supplement: Supplementary file 1 [file biomolecules-10-00519-s001.pdf]

Activins as dual specificity TGF-β family molecules: SMAD-activation via activin- and BMP-type I receptors

Olsen OE, *et al.*

Supplementary Material

Figure S1. Effects of knockdown of activin/TGF-β type 1 receptors in INA-6 myeloma cells
Figure S2. Effects of ALK2 knockdown in HepG2 cells
Figure S3. Activin-induced SMAD activity was not caused by an autocrine TGF-β loop

Table S1. Approved HUGO gene names for TGF-β family receptors
Table S2. TGF-β/SMAD inhibitors; reported inhibitory potential

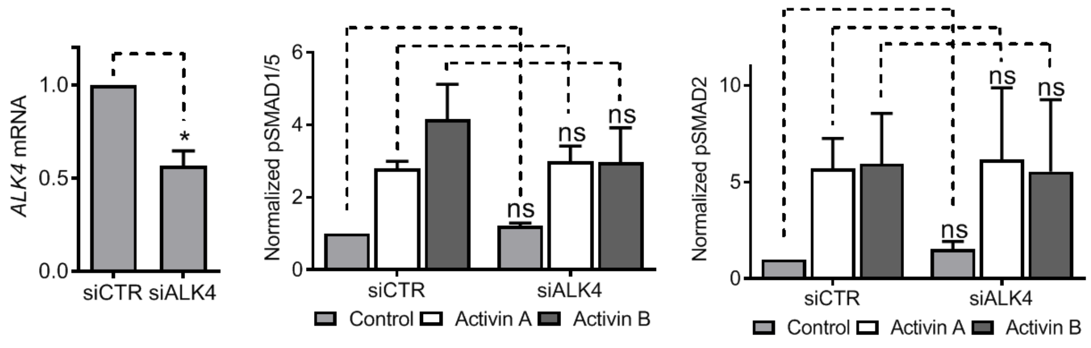

(**a**)

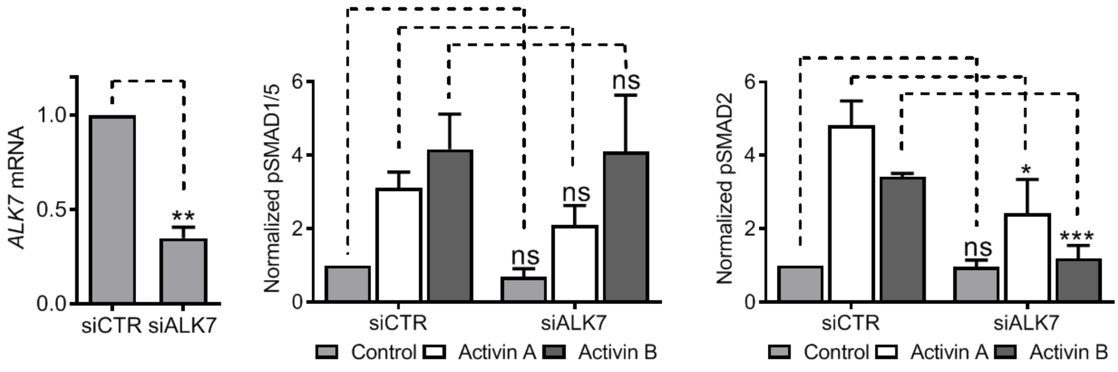

(**b**)

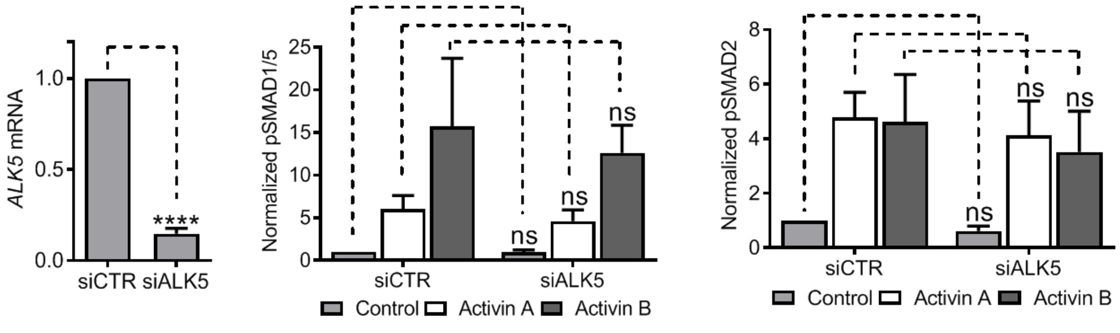

(**c**)

Figure S1. Effects of knockdown of activin/TGF-β type 1 receptors in INA-6 myeloma cells. To check for a possible involvement of the activin/TGF-β type 1 receptors ALK4, ALK7, or ALK5 in activin-induced activation of the two different SMAD branches, we did transient knockdown with siRNA targeting these receptors in INA-6 myeloma cells. The cells were treated 2 days post transfection with activin A (50 ng/mL) or activin B (10 ng/mL) for 2 hours. ALK4, ALK7 or ALK5 mRNA levels (**a-c**, left) were measured by PCR using the comparative Ct method and *GAPDH* as housekeeping gene. The graph represents mean±s.e.m. of n=3 independent experiments. Two-tailed, paired *t*-test was performed (*P≤0.05, **P≤0.01, ****P≤0.0001). The effect of reduced ALK4, ALK7, or ALK5 mRNA on relative activin A- or activin B-induced activation of SMAD1/5 (**a-c**, middle) or SMAD2 (**a-c**, right) was calculated based on signal intensities of the SMADs and GAPDH for normalization. The graphs represent mean±s.e.m. of n=3 independent experiments. Two-way ANOVA, Bonferroni's multiple comparisons test was performed (*P≤0.05, ***P≤0.001, ns (not significant) P>0.05).

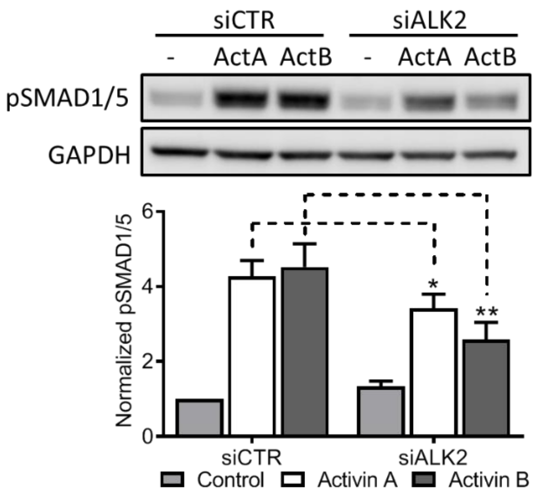

(**a**)

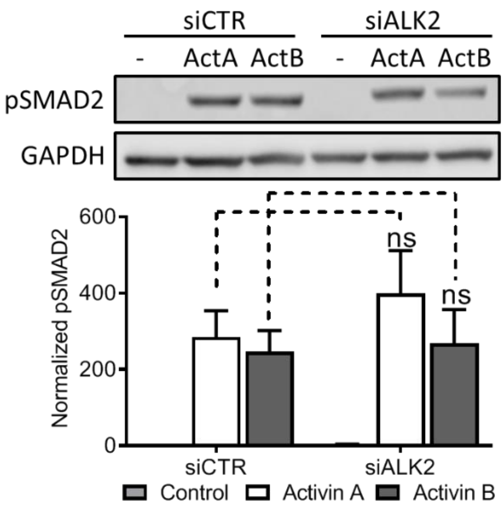

(**b**)

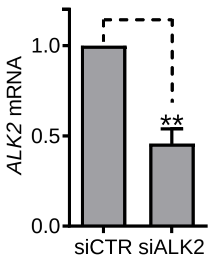

(**c**)

Figure S2. Effects of ALK2 knockdown in HepG2 cells. To check for a possible involvement of the BMP type 1 receptor ALK2 in activin-induced activation of the two different SMAD branches, we did transient knockdown with siRNA targeting ALK2 in HepG2 cells. The cells were treated the day after transfection with activin A (20 ng/mL) or activin B (60 ng/mL) for 1 hour. The effect of reduced ALK2 mRNA on relative activin A- or activin B-induced activation of SMAD1/5 (**a**) or SMAD2 (**b**) was calculated based on signal intensities of the SMADs and GAPDH for normalization. The graphs represent mean±s.e.m. of n=5 independent experiments. Two-way ANOVA, Bonferroni's multiple comparisons test was performed (*P≤0.05, **P≤0.01, ns (not significant) P>0.05). (**c**) ALK2 mRNA levels were measured by PCR using the comparative Ct method and *GAPDH* as housekeeping gene. The graph represents mean±s.e.m. of n=5 independent experiments. A two-tailed, paired *t*-test was performed (**P≤0.01).

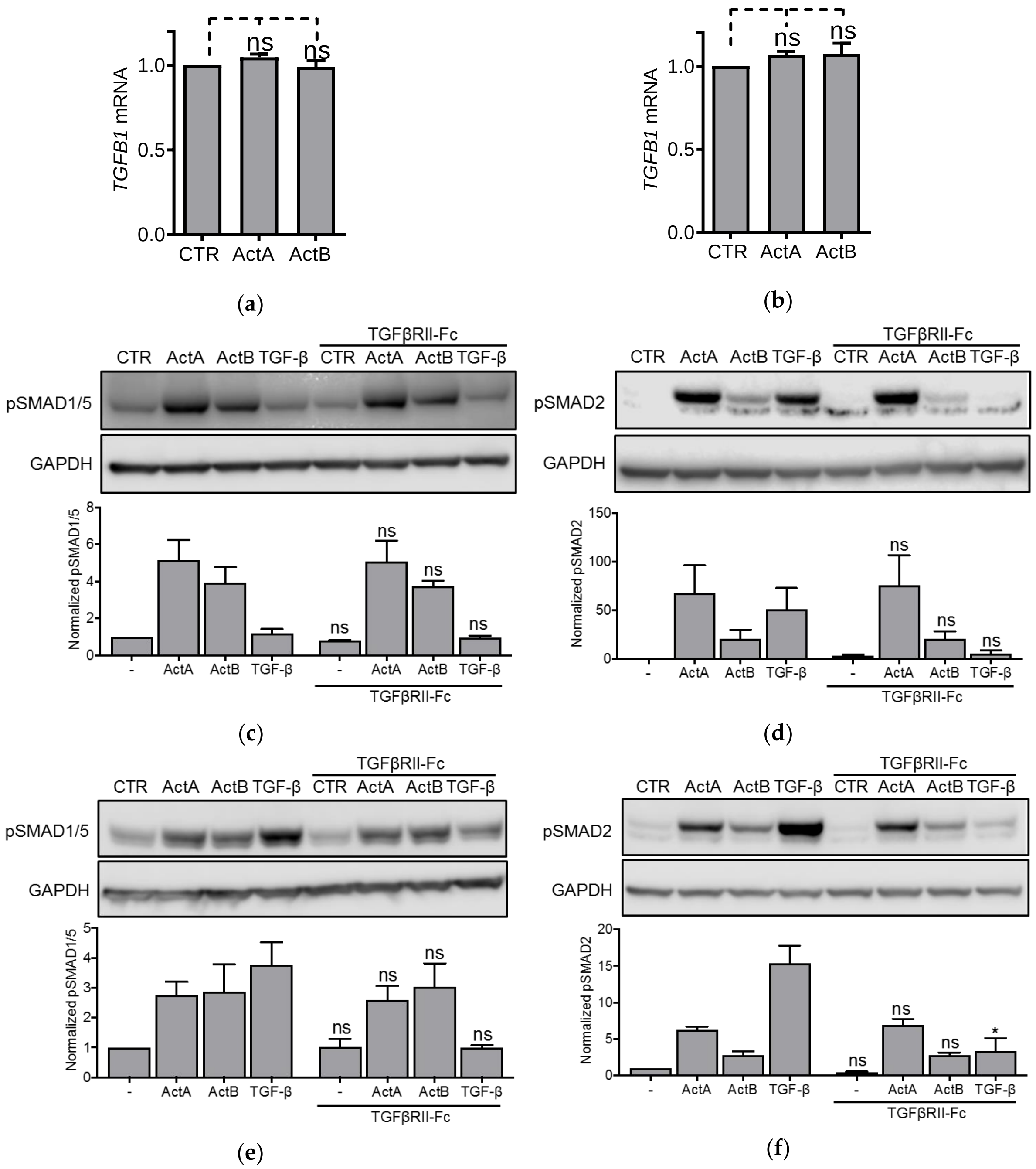

Figure S3. Activin-induced SMAD activity was not caused by an autocrine TGF-β loop. IH-1 (**a**) and INA-6 (**b**) cells were treated with activin A (20 ng/mL for IH-1 and 50 ng/mL for INA-6) and activin B (4 ng/mL for IH-1 and 10 ng/mL for INA-6) for 4 h and *TGFB1* mRNA was measured by PCR using the comparative Ct method and *GAPDH* as housekeeping gene. The graphs represent mean±s.e.m. of n=3 independent experiments. One-way ANOVA, Dunnett's multiple comparisons test was performed (ns (not significant) P>0.05). IH-1 cells were treated for 1 h with activin A (20 ng/mL), activin B (4 ng/mL) or TGF-β (5 ng/mL) with or without soluble TGFβRII-Fc (10 μg/mL) to look for a possible contribution of TGF-β activity on activation of SMAD1/5 (**c**) or SMAD2 (**d**). INA-6 cells were treated for 1 h with activin A (50 ng/mL), activin B (10 ng/mL) or TGF-β (0.5 ng/mL) with or without soluble TGFβRII-Fc (10 μg/mL) to look for a possible contribution of TGF-β activity on activation of SMAD1/5 (**e**) or SMAD2 (**f**). The graphs represent mean±s.e.m. of n=3 independent experiments. Two-way ANOVA, Bonferroni's multiple comparisons test was performed (*P≤0.05, ns (not significant) P>0.05).

Table S1. Approved HUGO gene names for TGF-β family receptors

Alias name	Approved symbol	Approved name
ALK1	ACVRL1	activin A receptor like type 1
ALK2	ACVR1	activin A receptor type 1
ALK3	BMPR1A	bone morphogenetic protein receptor type 1A
ALK4	ACVR1B	activin A receptor type 1B
ALK5	TGFBR1	transforming growth factor beta receptor 1
ALK6	BMPR1B	bone morphogenetic protein receptor type 1B
ALK7	ACVR1C	activin A receptor type 1C
ActRIIA	ACVR2A	activin A receptor type 2A
ActRIIB	ACVR2B	activin A receptor type 2B
BMPRII	BMPR2	bone morphogenetic protein receptor type 2
TGFβRII	TGFBR2	transforming growth factor beta receptor 2
AMHRII	AMHR2	anti-Mullerian hormone receptor type 2

Gene symbols and names of TGF-β family receptors as approved by the HUGO gene nomenclature committee (HGNC, https://www.genenames.org/).

Table S2. TGF-β/SMAD inhibitors; reported inhibitory potential.

	BMP Type 1 receptors				Activin/TGF-β Type 1 receptors		
**Name**	**ALK1**	**ALK2**	**ALK3**	**ALK6**	**ALK4**	**ALK7**	**ALK5**
K02288	++++	++++	+++	++++	++		++
ML347	+++	+++	-				
LDN-193189		++++	+++	++	++		++
SB431542					+	+	++
RepSox							++++
ZC-47-C95	-	-	-	-			++

The information on IC50 values for K02288, ML347, LDN-193189, SB431542, and RepSox was retrieved from various sources.(1-5) ZC-47-C95 was resynthesized compound 18a (6) retrieved from Novartis. "+" indicates IC50 values between 1-10 μM, "++"; between 0.1-1 μM, "+++"; between 0.01-0.1 μM, "++++"; between 0.001-0.01 μM, and "-" >10 μM.

References

1. Vogt, J., Traynor, R., and Sapkota, G. P. (2011) The specificities of small molecule inhibitors of the TGFss and BMP pathways. *Cell Signal* **23**, 1831-1842
2. Inman, G. J., Nicolas, F. J., Callahan, J. F., Harling, J. D., Gaster, L. M., Reith, A. D., Laping, N. J., and Hill, C. S. (2002) SB-431542 is a potent and specific inhibitor of transforming growth factor-beta superfamily type I activin receptor-like kinase (ALK) receptors ALK4, ALK5, and ALK7. *Molecular pharmacology* **62**, 65-74
3. Gellibert, F., Woolven, J., Fouchet, M. H., Mathews, N., Goodland, H., Lovegrove, V., Laroze, A., Nguyen, V. L., Sautet, S., Wang, R., Janson, C., Smith, W., Krysa, G., Boullay, V., De Gouville, A. C., Huet, S., and Hartley, D. (2004) Identification of 1,5-naphthyridine derivatives as a novel series of potent and selective TGF-beta type I receptor inhibitors. *Journal of medicinal chemistry* **47**, 4494-4506
4. Engers, D. W., Frist, A. Y., Lindsley, C. W., Hong, C. C., and Hopkins, C. R. (2013) Synthesis and structure-activity relationships of a novel and selective bone morphogenetic protein receptor (BMP) inhibitor derived from the pyrazolo[1.5-a]pyrimidine scaffold of dorsomorphin: the discovery of ML347 as an ALK2 versus ALK3 selective MLPCN probe. *Bioorganic & medicinal chemistry letters* **23**, 3248-3252
5. Sanvitale, C. E., Kerr, G., Chaikuad, A., Ramel, M. C., Mohedas, A. H., Reichert, S., Wang, Y., Triffitt, J. T., Cuny, G. D., Yu, P. B., Hill, C. S., and Bullock, A. N. (2013) A new class of small molecule inhibitor of BMP signaling. *PLoS One* **8**, e62721
6. Jin, C. H., Sreenu, D., Krishnaiah, M., Subrahmanyam, V. B., Rao, K. S., Nagendra Mohan, A. V., Park, C. Y., Son, J. Y., Son, D. H., Park, H. J., Sheen, Y. Y., and Kim, D. K. (2011) Synthesis and biological evaluation of 1-substituted-3(5)-(6-methylpyridin-2-yl)-4-(quinoxalin-6-yl)pyrazoles as transforming growth factor-beta type 1 receptor kinase inhibitors. *Eur J Med Chem* **46**, 3917-3925
